# Pharmacokinetic Evaluation of Eltrombopag in ITP Pediatric Patients

**DOI:** 10.3389/fphar.2021.772873

**Published:** 2021-12-06

**Authors:** Marco Dionisi, Sara Cairoli, Raffaele Simeoli, Francesca De Gennaro, Valeria Paganelli, Roberto Carta, Francesca Rossi, Carlo Dionisi-Vici, Giuseppe Palumbo, Bianca Maria Goffredo

**Affiliations:** ^1^ National Center for Drug Research and Evaluation, National Institute of Health (ISS), Rome, Italy; ^2^ Department of Pediatric Specialties and Liver-kidney Transplantation, Division of Metabolic Biochemistry, Bambino Gesù Children’s Hospital, IRCCS, Rome, Italy; ^3^ Department of Systems Medicine, University of Rome “Tor Vergata”, Rome, Italy; ^4^ Department of Pediatric Hemato-Oncology and Cell and Gene Therapy, Scientific Institute for Research and Healthcare, Bambino Gesù Children’s Hospital, IRCCS, Rome, Italy; ^5^ Department of Woman, Child and General and Specialist Surgery, University of Campania “Luigi Vanvitelli”, Naples, Italy

**Keywords:** eltrombopag, primary immune thrombocytopenia (ITP), pediatric patients, LC-MS/MS, pharmacokinetic (PK) evaluation, area under the concentration–time curve (AUC), gender and age influence, drug exposure

## Abstract

**Background:** Eltrombopag (EPAG) is an oral thrombopoietin receptor agonist, approved for refractory primary immune thrombocytopenia (ITP) in pediatric patients. In two pediatric RCTs, EPAG led to an improvement of platelet counts and a reduction in bleeding severity. However, a significant number of pediatric patients did not achieve the primary endpoints. We performed a pharmacokinetic evaluation of EPAG in pediatric patients with refractory ITP.

**Methods:** Outpatients aged from 1 to 17 y, affected by refractory ITP to first-line treatment, were enrolled for a pharmacokinetic assessment. The analysis of drug plasma concentration was performed by the LC-MS/MS platform. Non-compartmental and statistical subgroup analyses were carried out using the R package ncappc.

**Results:** Among 36 patients eligible for PK analysis, the median dose of EPAG given once daily was 50 mg. The EPAG peak occurs between 2 and 4 h with a population Cmax and AUC 0–24 geo-mean of 23, 38 μg/ml, and 275, 4 µg*h/mL, respectively. The pharmacokinetic profile of EPAG did not show a dose proportionality. Female patients showed a statistically significant increase of dose-normalized exposure parameters, increasing by 110 and 123% for Cmax and AUC 0–24, respectively, when compared to male patients. Patients aged 1–5 y showed values increased by more than 100% considering both exposure parameters, compared to older children. Furthermore, patients presenting complete response (83%), showed augmented EPAG exposure parameters compared to subjects with partial or no response.

**Conclusion:** These data highlight the need to further explore the variability of EPAG exposure and its pharmacokinetic/pharmacodynamic profile in pediatric patients also in a real-life setting.

## Introduction

Immune thrombocytopenia (ITP) is a hematological disease characterized by a low platelet count with an annual incidence of 2–5 cases/100,000 people (Marieke Schoonen et al., 2009). Specifically, diagnosis of ITP is established in the presence of a platelet count below 100 × 10^9^/L and in the absence of other possible causes of thrombocytopenia (Rodeghiero et al., 2009). Most patients with ITP are asymptomatic or present episodes of mucocutaneous bleeding at low degree; however, almost 15% of them require hospitalization due to bleeding within 5 y from diagnosis of the disease (Cooper and Ghanima, 2019). Pathogenesis of ITP is not fully understood; it is considered an auto-immune disease due to the formation of antibodies against platelets mediated by auto-reactive B cells; however, in recent years other pathological mechanisms have been proposed including an hyper-activation of the complement system, massive polarization toward a Th1 subtype of T cells, and a prevalence of the M1 pro-inflammatory macrophage phenotype with consequent production of pro-inflammatory cytokines (Johnsen, 2012; Consolini et al., 2016). The result of this immune deregulation is an increased platelet opsonization mediated by the monocyte/macrophage axis, which binds the Fc receptor of auto-reactive IgG and induces the elimination of platelets mainly in the spleen rather than the liver (He et al., 1994).

The spread of ITP within the pediatric population has been summarized by several epidemiological studies that report an ITP incidence comprised between 2.2 and 5.3 cases/10^5^ children *per* year (Fogarty and Segal, 2007; Terrell et al., 2010). Compared to adults, pediatric patients with ITP show a higher rate of spontaneous remission and a lower rate of co-morbidities (Schifferli et al., 2018). Moreover, among adult patients, there is a higher prevalence of ITP in female subjects than male subjects with a 2:1 ratio (Vianelli et al., 2001; Schifferli et al., 2018). This gender difference is less evident among pediatric ITP patients; however, in these subjects, a higher rate of severe bleeding has been reported compared to adults (Kühne et al., 2011).

Pharmacological treatment of pediatric patients with a new diagnosis of ITP includes corticosteroids and intra-venous immunoglobulins. This initial therapy, especially with steroids, should be suspended as soon as a safety level of platelet count (>20 × 10^9^/L) has been reached. Treatment of pediatric patients with persistent/chronic ITP consists mainly of thrombopoietin receptor (TPO-R) agonists, such as eltrombopag (EPAG) and romiplostim, the anti-CD20 monoclonal antibody rituximab, and mycophenolic acid (MMF). EPAG belongs to the family of biaryl-hydrazones and acts as a TPO-R agonist interacting with the subunit H499 present in the binding site of the TPO receptor. This interaction leads to a conformational change of the TPO-R structure that induces receptor activation and culminates in the final recruitment of the JAK/STAT cascade pathway. The pharmacological effect of EPAG interactions with TPO-R results in an increase of differentiation and proliferation rate of the megakaryocytoblastic cellular line and its precursors. Alongside this effect on platelet count, recent evidence also suggests an immune-modulating and iron chelating property of EPAG (Argenziano et al., 2021; Di Paola et al., 2021).

Safety and efficacy of EPAG have been evaluated in pediatric patients with two registration studies: TRA115450 (PETIT2) and TRA108062 (PETIT) (Bussel et al., 2015; Grainger et al., 2015). In both studies, the primary endpoint was the percentage of patients who reach a platelet count ≥50,000/µl at least once during the randomized therapeutic period. In the same studies, pharmacokinetic (PK) properties of EPAG have been evaluated using a population model on 168 pediatric patients with ITP who were assuming EPAG once a day by oral administration. The results of these studies show that following oral dosage, plasmatic clearance of EPAG proportionally increases with body weight and that female pediatric patients with ITP show a 25% increase of the area under the curve (AUC) of EPAG plasmatic concentrations compared to male pediatric patients (Wire et al., 2018).

EPAG PK data reported in the literature are based on pharmacokinetic population models, reliant on data deriving from randomized clinical trials, where patients are enrolled and strictly monitored throughout the study. Here, however, we present the results of EPAG PK evaluation performed on pediatric patients affected by ITP treated with EPAG, including “real-life” data collected during routine clinical practice.

## Materials and Methods

### Study Design and Procedures

This study was conducted at the Department of Pediatric Hematology and Oncology, Cell and Gene Therapy at Children’s Hospital Bambino Gesù in Rome, from December 2019 to March 2021. All procedures were included in a therapeutic drug monitoring application routinely performed in our hospital. Outpatients were included if aged from 1 to 17 y, with a confirmed diagnosis of primary immune thrombocytopenia, had relapsed or refractory disease after one or more previous treatments for immune thrombocytopenia, and were orally treated with EPAG film-coated tablets (Revolade®, Novartis Europharm Limited, Dublin, Ireland) for at least 2 weeks without dose variations. Patients who had other significant non-ITP-related diseases were excluded. Following hospitalization, all patients or legal guardians gave their written informed consent to receive diagnostic procedures and pharmacological treatments. Thereafter, a verbal informed consent was asked to all patients or legal guardians before study procedures. The study was conducted in accordance with the Declaration of Helsinki principles. The ethical committee of our hospital was informed about this therapeutic drug monitoring application.

Enrolled outpatients were present in the clinic from the morning of the visit day for a maximum of 8 h. In addition to routine laboratory analyses and medical exams and procedures, blood samples (3 ml) were obtained through an indwelling peripheral catheter and collected into vacutainer tubes containing EDTA (Becton Dickinson, Rutherford, NJ, United States) immediately before the dose and at 2, 4, and 8 h after the dose. Whenever possible, blood was obtained from central laboratory specimens. Additional or alternative time sampling was allowed in accordance with the ward sampling routine. Blood drawing was performed in accordance with study-related blood loss limits indicated by the EU Commission (European Union, 2008). Routine laboratory blood tests included hematology and chemistry analyses. At the time of the visit, qualified medical staff performed a complete physical exam, also collecting information about ITP duration, response, adverse events, and patients’ compliance. Moreover, patients or legal guardians were provided with a diary-like form to fill out the day of the visit. The form contained information such as time of dose administration the day before the visit, concomitant medications, smoking habits (for adolescents), consumption of dairy products, and mineral supplements or caffeinated drinks along with meal times.

A complete response (CR) was defined as at least a single value of PLT >100,000/µl 1 week after starting EPAG therapy, without the need of rescue therapy and no signs of bleeding. A partial response (PR) was characterized by a value of PLT between 30,000/µl and 100,000/µl, and no response (NR) was defined as a number of PLT <30000/µl. A durable response (DR) consists in detection of PLT >50,000/µl for at least 75% of the follow-up period after the initiation of EPAG treatment. Rescue therapy included corticosteroids and/or intravenous immunoglobulins (IVIg). ITP duration was classified according to disease duration since diagnosis: newly diagnosed ITP (<3 months), persistent ITP (6–12 months), and chronic ITP (>12 months) (Rodeghiero et al., 2009).

### Drug Assay

Plasma was obtained by centrifuging at 3,500 g for 5 min. 50 µL of plasma was added to 25 µL of IS working solution (50 μg/ml); then, 250 µL of methanol was used to precipitate proteins; samples were vortex-mixed for 30 s and centrifuged at 13,000 rpm for 9 min. The supernatant (10 µl) was then injected into the UHPLC-MS/MS system for analysis. A standard stock solution was prepared dissolving EPAG in methanol and 0.1% of dimethyl sulfoxide (DMSO) at a concentration of 1 mg/ml. The calibration standard solutions were prepared spiking standard stock solutions to drug-free plasma at the following concentrations: 1, 5, 10, 25, 50, 100, and 150 μg/ml. Quality controls were prepared by serial dilution of the stock solution with drug-free plasma at different concentrations: QC low 7.5 μg/ml, QC medium 15 μg/ml, and QC high 75 μg/ml. The stock solution of IS (1 mg/ml) was prepared by dissolving EPAG 13C4 in DMSO. A working solution of the internal standard (50 μg/ml) was prepared in methanol.

Chromatography analysis was performed using a UHPLC Agilent 1290 Infinity II (Agilent Technologies). The separation column was a Kinetex 2.6 μm EVO C18 100 Å 75 × 2.1 mm (Phenomenex, Torrance, CA). Mobile phase A was water with 0.1% formic acid, 31% acetonitrile, and 31% methanol v/v, and mobile phase B was methanol with 0.5% formic acid v/v. The flow rate was 0.3 ml/min according to the following gradient: 0–2.9 min 55% B, 3–3.1 min 95% B, 3.1–4.0 min 95% B, and 4.1–5.0 min 55% B. The injection volume was 10 μl. Samples were analyzed with a 6,470 mass spectrometry system (Agilent Technologies) equipped with an ESI-JET-STREAM source operating in a positive ion (ESI+) mode. The software used for controlling this equipment and analyzing results was MassHunter (Agilent Technologies). Samples were detected in the multiple reaction monitor (MRM) mode. The mass transitions of EPAG were m/z 443.2→ 183 for the quantifier and 443.2 →77 for the qualifier.

All chemicals were of analytical grade and were obtained from Sigma-Aldrich (Saint Louis, MO, United States). EPAG and EPAG-13C4 (employed as an internal standard) were obtained from Toronto Research Chemicals (Toronto, Canada). Drug-free plasma was obtained from healthy volunteers recruited at the Blood Transfusion Center of the Children’s Hospital Bambino Gesù after obtaining informed consent and was used as a matrix for standard curve preparation and negative controls. Method validation was performed based on the US Food and Drug Administration (FDA) guidelines for industry bioanalytical method validation. Method validation was carried out including specificity, linearity, inter- and intra-precision and accuracy, extraction recovery, and the matrix effect (data not shown).

### Pharmacokinetic Analysis

Analysis of plasma concentration–time data was conducted using the noncompartmental approach using the ncappc package (v0.3.0; Acharya et al., 2016) and Rstudio (RStudio: Integrated Development for R, 2020. RStudio, Inc., Boston, MA URL http://www.rstudio.com/). The estimated pharmacokinetic parameters of EPAG were area under the plasma concentration–time curve from time zero to 24 h (AUC 0-24), maximum observed plasma concentration (Cmax), and time to reach Cmax (Tmax). The area under the concentration–time curve from 0 to 24 h post dose (AUC 0-24) was assessed using the linear-log trapezoidal rule from zero up to the 24 estimated concentration. Dose normalized values (AUC 0-24 DN and Cmax, DN) were derived by dividing AUC 0-24 and Cmax by dose. The concentration at 24 h was estimated using the equation (C24h) = C8h * e ^*-β(t24–t8)*^ if the concentration at 8 h post dose was quantifiable (Ruslami et al., 2007).

### Statistical Analysis

Demographic data and pharmacokinetic parameters were summarized using descriptive statistics. No formal power calculation was made for the study. Mean with standard deviation or median with ranges was used for normally distributed and non-normally distributed variables, respectively. Number of occurrence and percentage were used to describe categorical data. Cmax, AUC 0-24, and dose-normalized pharmacokinetic parameters were described through the geometric mean and 95% confidence intervals. EPAG dose proportionality over the dose range of 12.5–100 mg was evaluated using a power model, assuming a linear relationship between natural log-transformed exposure parameters (Cmax and AUC 0-24) and natural log-transformed dose values: *ln(PK parameter) = β0 + β1-ln(dose)*. Furthermore, an equivalence criterion was also used to evaluate the inclusion of the proportional constant β1 and its 90% confidence interval within the acceptance range Food and Drug Administration (FDA) and Center for Drug Evaluation and Research (CDER), (2001). Guidance for Industry: Statistical Approaches to Establishing Bioequivalence. The maximal dose ratio (r) was 8 (100/12.5). The estimated lower limit {*1+* [ln*(0.8)/*ln*(r)*]} and the upper limit {*1+* [ln*(1.25)/*ln*(r)*]} were 0.892 and 1.107, respectively. The unpaired *t*-test and one-way ANOVA were used to compare log-transformed exposure parameters taking into account the ITP duration and response. Dose-normalized log-transformed pharmacokinetic exposure parameters were considered to compare gender, age groups, and ITP duration. All statistical analyses and graphs were performed using Rstudio (RStudio: Integrated Development for R, 2020. RStudio, Inc., Boston, MA URL http://www.rstudio.com/).

## Results

### Patients

The study comprised 36 patients, 61 percent of which were females (*n* = 22). The median age was 10.94 years (range 2.67–17.9 years), and the median body weight was 45.4 kg (range 15.9–109 kg), as shown in Table 1. The dose of EPAG given once daily ranged between 12.5 (*n* = 1) and 100 mg (*n* = 3). Twelve patients were administered 75 mg of EPAG, and the same number of patients were treated with 50 mg. Eight subjects were treated with 25 mg. The mean dose for all patients was 55.9 mg (standard deviation: 24.0 mg). Mean dose values were similar between the two younger age groups, namely, 1–5 y and 6–11 y. Instead, older children were treated with higher doses (mean = 64.8 mg). The median platelet count was 86.5 PLT x 10^9^/L, ranging from 10 to 1611 PLT x 10^9^/L. The percentage of patients with a diagnosis of newly diagnosed ITP (ND), persistent ITP (P), or chronic (C) ITP was similar in the study population; nonetheless, the 6–11 y age group showed a higher frequency of ND. Additionally, more than half of the patients affected by P or C ITP were among the 12–17 y age group. Overall, a complete response was found in 30/36 patients (83%). However, only 33% of patients showed a durable response. Rescue therapy during EPAG treatment was required in five patients (13.9%), mainly in the 1–5 y age group.

**TABLE 1:** Patients’ characteristics at the time of the study visit and estimated pharmacokinetic parameters.

	All	1–5 years	6–11 years	12–17 years
Patients, n (%)	36 (100%)	8 (22.2%)	12 (33.3%)	16 (44.5%)
Age (years), median	10.94	5.08	8.44	15.08
Gender (F) n (%)	22 (61.1%)	6 (27.3%)	5 (22.7%)	11 (50%)
Dose (mg) Mean (SD)	55.9 (24.0)	50.0 (13.3)	47.9 (22.5)	64.8 (27.0)
Body Weight (kg), Median (range)	45.43 (15.5–109)	19.1 (15.5–28.5)	31.5 (20.0–60.0)	60.0 (37.0–109.0)
Platelet count (x 10^9^/L), Median (range)	86.5 (10–1,611)	101.5 (10–1,307)	105.5 (17–1,611)	86.5 (24–235)
ITP Duration, n (%)				
Newly Diagnosed	12 (33.3%)	4 (33.3%)	6 (50%)	2 (16.7%)
Persistent	13 (36.1%)	3 (23.1%)	3 (23.1)	7 (53.8%)
Chronic	11 (30.6%)	1 (9.1%)	3 (27.3%)	7 (63.6%)
Response, n (%)				
Complete	30 (83.3%)	6 (20%)	11 (36.7%)	13 (43.3%)
Partial	4 (11.1%)	1 (25%)	-	3 (75%)
Absent	2 (5.6%)	1 (50%)	1 (50%)	-
Durable Response, n (%)	12 (33.3%)	1 (8.3%)	4 (33.3%)	7 (58.4%)
Rescue therapy, n (%)	5 (13.9%)	4 (80%)	-	1 (20%)
T max, h mean (SD)	2.62 (0.90)	2.52 (0.91)	2.37 (0.77)	2.85 (0.94)
Cmax, µg/mL geo-mean (CI 95%)	23.38 (16.20–33.74)	43.00 (26.60–69.66)	18.29 (9.25–36.19)	20.71 (11.63–36.89)
AUC 0–24, µg*h/mL geo-mean (CI 95%)	275.40 (185.87–408.02)	515.48 (278.28–954.88)	200.50 (108.42–970.78)	255.38 (131.19–497.14)
Cmax DN, µg/mL geo-mean (CI 95%)	0.47 (0.34–0.63)	0.89 (0.61–1.31)	0.43 (0.25–0.73)	0.362 (0.23–0.58)
AUC 0–24 DN, µg/mL geo-mean (CI 95%)	5.51 (3.94–7.69)	10.7 (6.17–18.52)	4.68 (2.83–7.72)	4.47 (2.57–7.76)

### Concentration–Time Profile of EPAG in Our Pediatric Population

**FIGURE 1:**
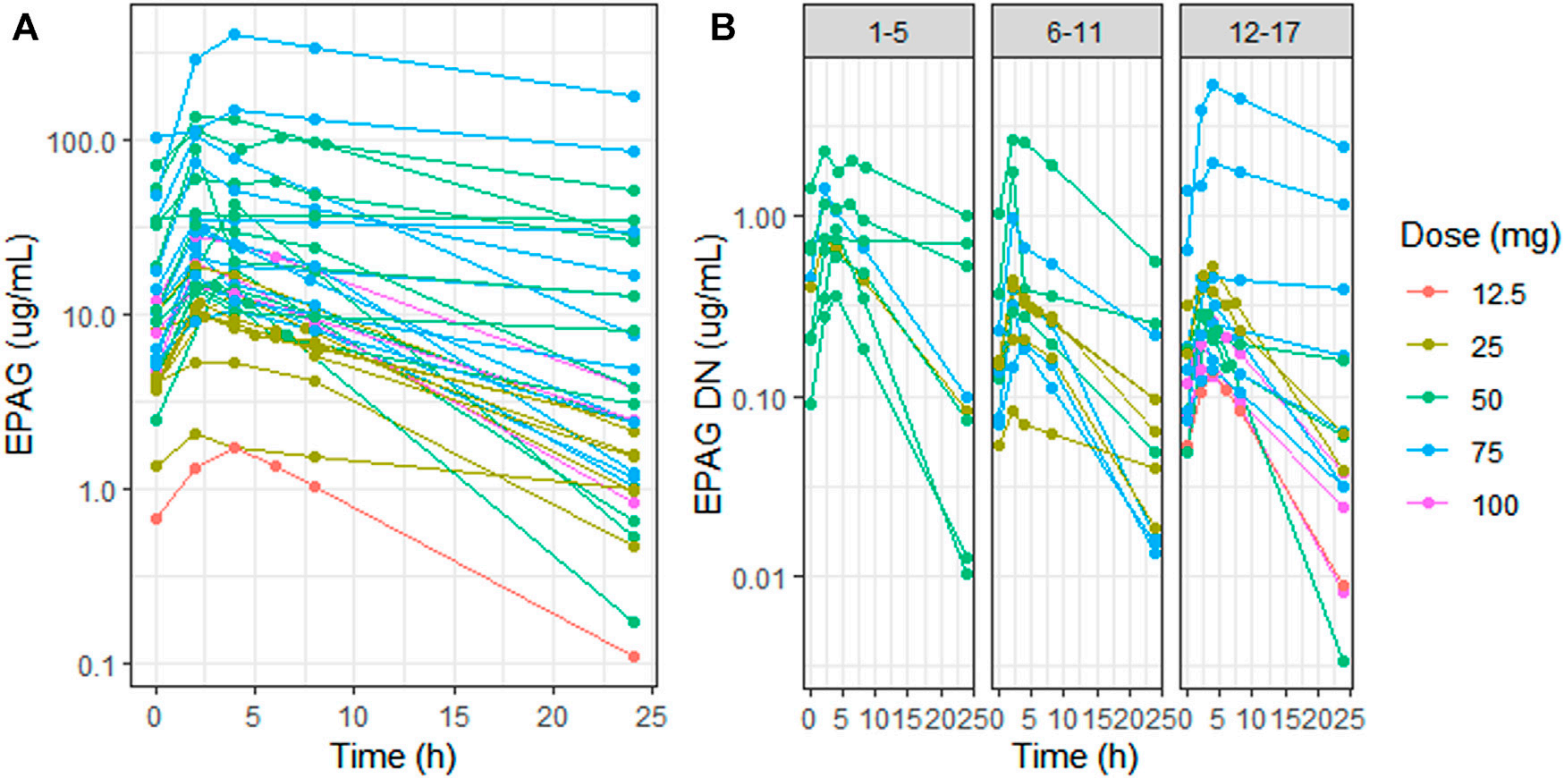
Eltrombopag concentration (log scale) vs. time in all patients **(A)** and dose-normalized eltrombopag concentration (log scale) vs. time in the three different age groups (1–5 y, 6–11 y, and 12–17 y) **(B)**. Patient profiles were stratified by dose: 12.5 mg (red), 25 mg (light-green), 50 mg (green), 75 mg (blue), and 100 mg (purple). EPAG: eltrombopag and DN: dose-normalized.

Marked inter-individual variability of the EPAG concentration–time profile was observed in the whole study population (CV% Cmax DN = 130%, CV% AUC 0–24 DN = 157%) (Figure 1A), with a high range of variability differences also among patients within the same age group despite the dose normalization of concentrations (CV% Cmax DN 1–5 y = 58%, 6–11 y = 114%, 12–17 y = 183%, CV% AUC 0–24 1–5 y = 80%, 6–11 y = 129%, and 12–17 y = 201%) (Figure 1B). Overall, the EPAG peak occurs between 2 and 4 h (mean = 2.62 h) with a Cmax and AUC 0–24 geo-mean of 23, 38 μg/ml (CI95% 16.20–33.74 μg/ml) and 275.4 µg*h/mL (CI95% 185.87–408.02 µg*h/mL), respectively. The dose-normalized Cmax and AUC 0-24 were 0.47 μg/ml and 5.51 µg*h/mL, respectively (Table 1).

### Evaluation of Dose Proportionality Following EPAG Oral Administration

**FIGURE 2:**
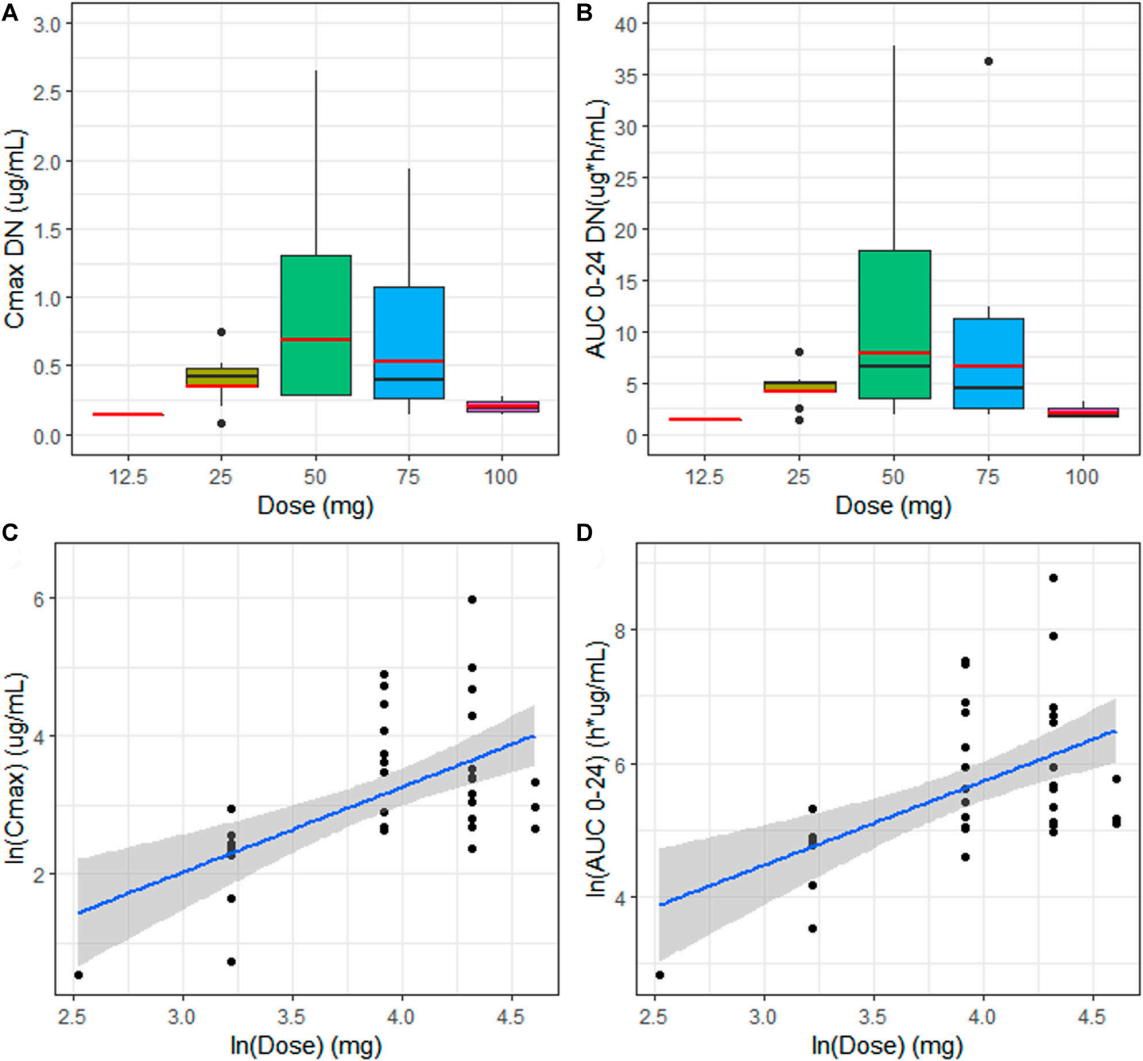
**(A, B)** Median (black line) and geo-mean (red line) of dose-normalized Cmax (Cmax DN) and dose-normalized AUC 0–24 (AUC 0–24 DN) vs. dose (mg) for the evaluation of dose proportionality. **(C)** Linear regression model with the best fit line (blue) and 90% CI (gray area) describing relationship between the extent of systemic exposure including Cmax and AUC 0–24 **(D)** and dose.

EPAG did not show a dose proportionality, considering both exposure parameters, with higher values of Cmax and AUC 0–24 in patients treated with 50 mg Figure 2(A,B). The estimates of the proportionality constant (90% CI) for Cmax and AUC 0-24 were 0.17 (0.09–0.24) and 0.15 (0.08–0.22), respectively Figure 2(C,D). Hence, according to the equivalence criterion limits, the proportional constant was not included within the prespecified acceptance limits (0.89–1.10) for dose proportionality.

### Influence of Gender and Age on EPAG Exposure

**FIGURE 3:**
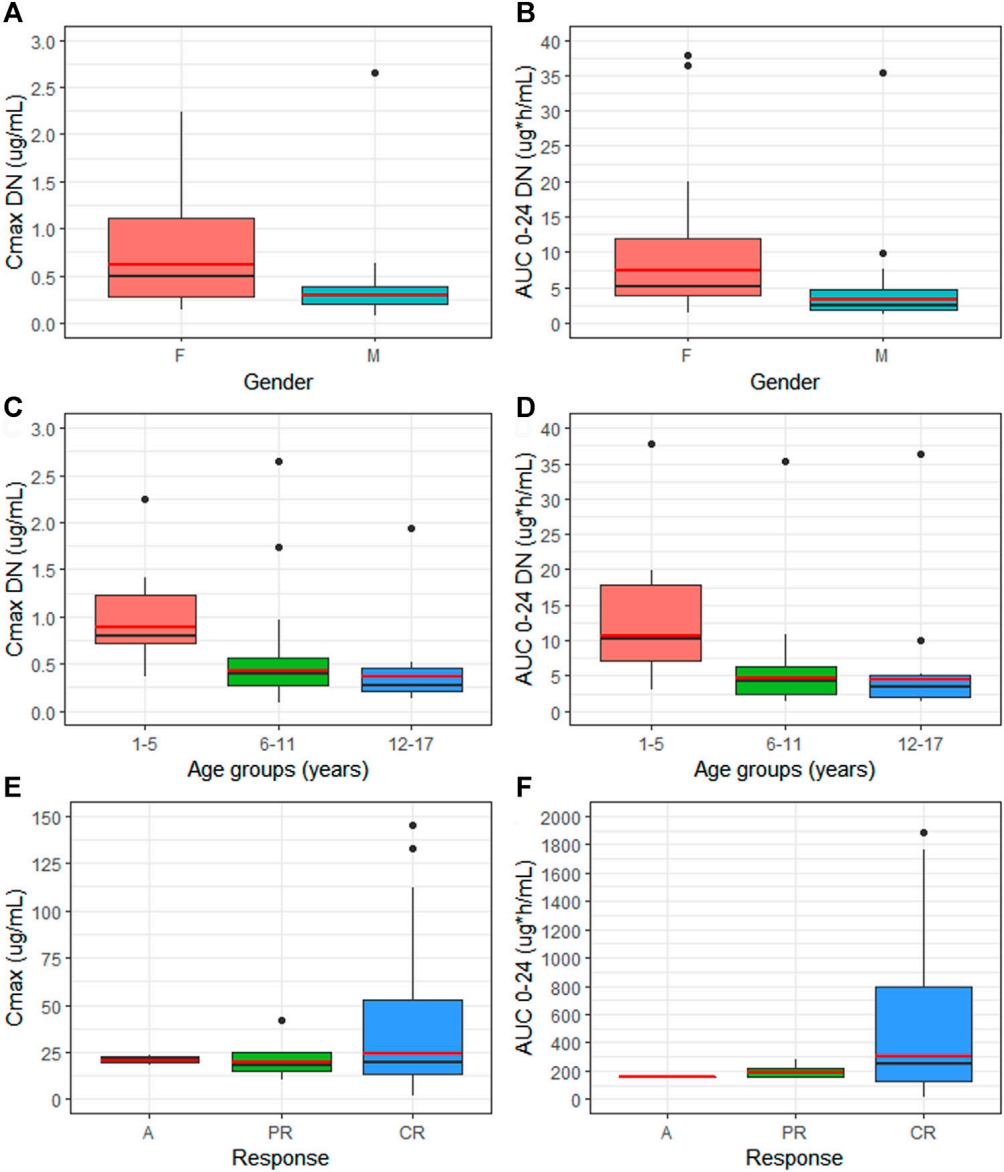
Comparison of median (black line) and geo-mean (red line) of dose-normalized Cmax (Cmax DN) and dose-normalized AUC 0–24 (AUC 0–24 DN) between gender **(A,B)** and different age groups **(C,D)**. Median (black line) and geo-mean (red line) of exposure parameters between patients with distinct EPAG response **(E,F)**. M = male, F = female, C = complete response, PR = partial response, A = no response.

Females showed a statistically significant increase of dose-normalized exposure parameters (Cmax DN = 0.619 μg/ml and AUC 0-24 DN = 7.48 µg*h/mL) when compared to males (Cmax DN = 0.301 μg/ml and AUC 0–24 DN = 3.40 µg*h/mL), with a percentage increase of 110 and 123% for Cmax and AUC 0–24, respectively (*p* = 00,201 and *p* = 00,198) Figure 3(A,B). No statistically significant differences in dose-normalized Cmax and AUC 0-24 were detected between the distinct age groups. Nevertheless, patients aged 1–5 y showed higher Cmax DN and AUC 0-24 DN (0.89 μg/ml and 10.7 µg*h/mL), compared to the other two groups, undergoing an increase of more than 100% for both exposure parameters Figure 3(C,D). Cmax DN and AUC 0–24 DN were similar between 6–11 y and 12–17 y age groups (Table 1).

### Differences in Cmax and AUC among Patients with Partial, Complete, and Durable Response to EPAG Therapy

Although not statistically significant, patients presenting a CR during EPAG treatment showed augmented values of Cmax and AUC 0-24 geo-mean (24.16 μg/ml, CI95% 15.6–37.3 μg/ml and 299.84 µg*h/mL, CI95% 188.23–477.63 µg*h/mL, respectively), compared to patients with PR (19.44 μg/ml, CI95% 11.0–34.2 μg/ml and 191.10 µg*h/mL, CI95% 144.08–253.49 µg*h/mL) or no response (20.67 μg/ml and 159.68 µg*h/mL) Figure 3(E,F). No differences in EPAG exposure were found when stratifying patients through evidence of durable response and the need of rescue therapy during EPAG treatment (Supplementary Figure S1). Moreover, as concerns adverse reactions during eltrombopag treatment, a higher percentage of patients (52.77%) did not experience side effects, followed by *n* = 6 (16.66%) patients who reported headaches and *n* = 4 (11.11%) who showed Grade 3 hypertransaminasemia. Exclusively in this latter situation, EPAG treatment was briefly suspended until clinical parameters were normalized (Supplementary Table S1).

## Discussion

The use of oral drugs acting on the thrombopoietin receptor has dramatically changed the therapeutic paradigm of first-line refractory and relapsed ITP patients. EPAG is an orally thrombopoietin receptor agonist already approved for ITP treatment in pediatric patients (Burness et al., 2016; Wong et al., 2017; Kim et al., 2018; Fattizzo et al., 2019). EPAG’s main mechanism of action is related to the stimulation of platelet production. However, it is also endowed with immune-modulating properties reducing Th1 activation (Bao et al., 2010) and inhibiting macrophage switch toward an inflammatory phenotype (Liu et al., 2016; Di Paola et al., 2021). In this study, we propose an exploratory pharmacokinetic evaluation of EPAG exposure in pediatric patients affected by ITP.

In particular, our data show a high degree of inter-variability of EPAG pharmacokinetic within the selected pediatric population. Moreover, our results reveal a lack of EPAG dose proportionality when analyzing the available data according to a power model with the equivalence acceptance criterion. Such evidence represents a novelty if compared to the already published results. In fact, several studies have reported a predictable and dose-proportionate PK behavior of EPAG in ITP patients (Gibiansky et al., 2011; Matthys et al., 2011; Wire et al., 2018). However, this discrepancy could be explained by the fact that our data derive from a “real-life” setting where patients are not hospitalized and, therefore, easily influenced by extrinsic factors such as different dietary habits and poor compliance. As a proof of this, although it was recommended to assume EPAG at least 2 h before or 4 h after meals, at the day of the visit, the overwhelming majority of patients (94%) did not follow the recommendation. For example, almost 17% of patients consumed caffeinated beverages 4 h following EPAG dose. In terms of concomitant medications, around 35% of patients were taking vitamins (Vitamin D, C, and B) and Mg^2+^/K^+^ saline integrators. Conversely, literature data are based on pharmacokinetic population models based on data extracted from randomized clinical trials, where patients are enrolled and strictly monitored throughout the study.

According to the literature, our data confirm that female patients show a higher drug exposure compared to male patients with an increase of 110 and 123% for Cmax DN and AUC 0-24 DN, respectively, compared to males. Conversely, no statistically significant differences in weight between the two genders were found (median body weight F = 43.5 kg, M = 42.95 kg). The gender influence on drug exposure has been well documented by Wire et al. (2018), who included sex as a covariate influencing EPAG clearance in pediatric subjects. These findings could be explained by a reduced metabolic activity of CYP1A2 in females (Relling et al., 1992; Parkinson et al., 2004). This cytochrome isoform is one of the two major enzymes involved in the oxidative hepatic metabolism of EPAG.

Together with gender, we have also analyzed the effect of age on EPAG exposure. Although not statistically significant, younger children (1–5 y age group) displayed increased dose normalized exposure PK values when compared to the children aged more than 6 years, potentially due to a lower body weight and the reduced hepatic EPAG metabolism (Salem et al., 2014; Song et al., 2017; Wire et al., 2018). Furthermore, despite the recommended starting EPAG dose being 25 mg in children aged 1–5 y, only one patient from this age group has been treated with this dose (mean dose 1–5 y age group = 50 mg).

As previously reported, EPAG treatment in pediatric patients reached a response rate between 40 and 81% (Bussel et al., 2015; Grainger et al., 2015; Neunert et al., 2016). In our study, 30 patients (83%) presented a CR characterized by higher Cmax and AUC 0–24 geo-means compared to PR or non-responder patients. Only 33% of the patients showed durable response. Remarkably, among the six patients with CR in the 1–5 y age group, four of them needed a rescue therapy during treatment. Taking into account the monocentric nature of the study and the incidence of ITP in the pediatric population, a limitation of this study is the relatively contained number of enrolled subjects. This could have impaired the comparison of exposure parameters between the groups. An additional limitation is the extrapolation of the concentration at a 24 h time point since the enrolled subjects were outpatients. However, we decided not to replicate the marketing authorization holder method (the pre-dose sample was duplicated and analyzed as a 24 h sample under the assumption that the PK steady state had been reached) taking into account the variability of the drug administration time the day before the visit in our study population (CHMP, Ema, 2011).

In conclusion, this study showed a marked variability and unpredictability of EPAG concentrations in pediatric outpatients affected by ITP. However, our findings confirmed the gender influence on EPAG exposure. Furthermore, although not statistically significant, children aged 1–5 y had increased dose-normalized PK exposure values compared to older patients. EPAG administration requires dose adjustment according to PLT count throughout treatment. Therefore, a better comprehension of PK behavior of this TPO-RA, particularly in a real-life setting, could shed light on exposure and response gaps in some patient subpopulations and could be a potential tool for dose optimization during EPAG treatment. Nevertheless, 1–5 y old patients resorted more frequently to rescue therapy, despite higher DN exposure EPAG parameters when compared to the other age groups. This suggests that the response to EPAG might not be as strongly linked to PK parameters. To sum up, these results highlight the need to further investigate the lack of complete response persistence and treatment-free ITP remission during and after EPAG treatment in pediatric patients.

## Data Availability

The raw data supporting the conclusion of this article will be made available by the authors, without undue reservation.
