# Supplementary material for: Pharmacokinetic Evaluation of Eltrombopag in ITP Pediatric Patients
Source: Front Pharmacol. 2021 Dec 6;12:772873. doi: 10.3389/fphar.2021.772873 (PMC8685423; doi:10.3389/fphar.2021.772873)
Supplement: Supplementary file 1 [file DataSheet1.doc]

Supplementary Material

**Supplementary Figure 1.** Comparison of median (black line) and geo-mean (red line) of Cmax and AUC 0-24 between patients who needed rescue therapy during EPAG treatment (**A-B**), and patients with evidence of durable response (**C-D**).


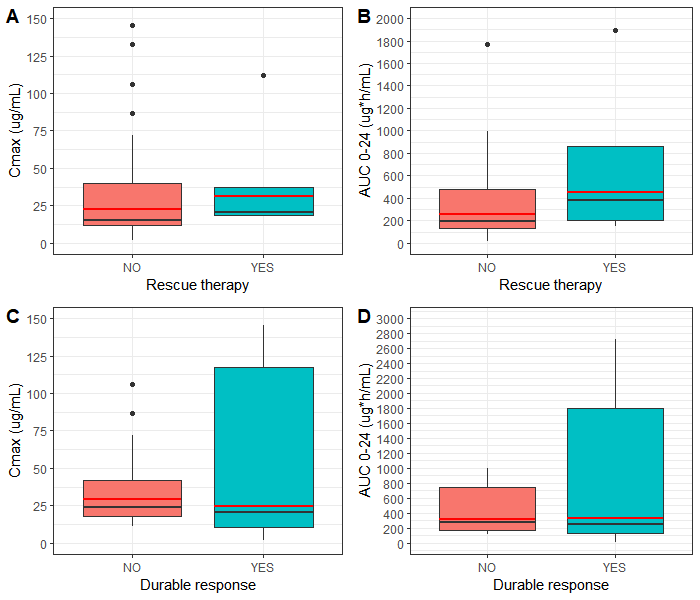


**Supplementary Table 1.** Adverse reactions reported during Eltrombopag treatment in our pediatric population (n=36 patients corresponding to 100% of total studied population).

| **Adverse Reaction** | **Patients,** n (%) | **Eltrombopag**  **suspension or dose reduction** |
| --- | --- | --- |
| **None** | 19 (52.77 %) | None |
| **Vomiting** | 1 (2.77 %) | None |
| **Headache** | 6 (16.66 %) | None |
| **Grade 2 hypertransaminasemia** | 1 (2.77 %) | Brief suspension |
| **Grade 3 hypertransaminasemia** | 4 (11.11 %) | Brief suspension |
| **Vomiting plus Headache** | 1 (2.77 %) | None |
| **Vomiting plus Grade 2 hypertransaminasemia** | 1 (2.77 %) | None |
| **Hyperalbuminemia plus Grade 3 hypertransaminasemia** | 1 (2.77 %) | Brief suspension |
| **Headache plus Grade 3 hypertransaminasemia** | 1 (2.77 %) | Brief suspension |
| **Headache and Vomiting plus Grade 3 hypertransaminasemia** | 1 (2.77 %) | Brief suspension |

**
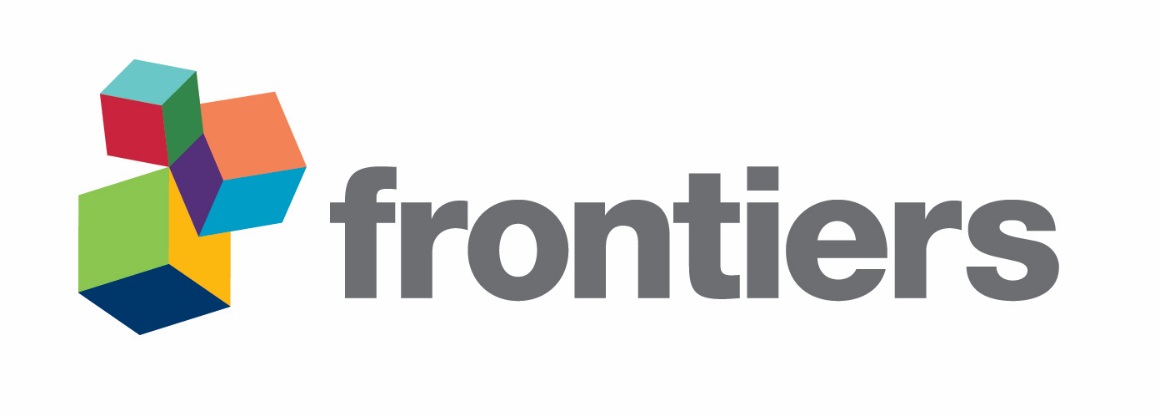
**
